# Pharmacological Inhibition of Inositol Hexakisphosphate Kinase 1 Protects Mice against Obesity-Induced Bone Loss

**DOI:** 10.3390/biology11091257

**Published:** 2022-08-24

**Authors:** Siddaraju V. Boregowda, Manjunatha K. Nanjappa, Cori N. Booker, Jacqueline Strivelli, Valentina M. Supper, Paul S. Cooke, Donald G. Phinney

**Affiliations:** 1 Department of Molecular Medicine, UF Scripps Biomedical Research, Jupiter, FL 33458, USA; 2 Department of Physiological Sciences, University of Florida, Gainesville, FL 32610, USA; 3 J. Crayton Pruitt Family Department of Biomedical Engineering, University of Florida, Gainesville, FL 32610, USA

**Keywords:** diet, high fat, obesity, osteoporosis, anti-obesity drugs, inositol hexakisphosphate kinase 1, stem cells, skeletal, mesenchymal stem cells

## Abstract

**Simple Summary:**

Obesity and diabetes have detrimental impacts on skeletal health that result in an increased fracture risk and impaired fracture healing, conditions associated with significant morbidity and mortality and that are costly to treat. Managing obesity-induced bone loss is complicated by the fact that anti-diabetic drugs have negative or unknown impacts on bone health, which limits their effectiveness. Inositol hexakisphosphate kinase 1 (IP6K1) functions as a prominent regulator of energy expenditure based on data showing its inhibition protects mice from diet-induced obesity. In this paper, we show that the pharmacological inhibition of IP6K1 after the onset of a high-fat diet feeding protects mice against weight gain and its associated metabolic derangements even under thermo-neutral conditions, and that kinase inhibition also preserves bone mass, bone micro-architecture, and the pool of skeletal stem/progenitors in bone marrow. Obesity also has negative impacts on male fertility, but prolonged IP6K1 inhibition had no adverse impacts on male reproductive parameters. These findings identify IP6K1 as a preferred target for glycemic control due to the bone sparing effects of IP6K1 inhibition.

**Abstract:**

Obesity and type II diabetes mellitus (T2DM) are prominent risk factors for secondary osteoporosis due to the negative impacts of hyperglycemia and excessive body fat on bone metabolism. While the armamentarium of anti-diabetic drugs is expanding, their negative or unknown impacts on bone metabolism limits effectiveness. The inactivation of inositol hexakisphosphate kinase 1 (IP6K1) protects mice from high-fat-diet (HFD)-induced obesity (DIO) and insulin resistance by enhancing thermogenic energy expenditure, but the role of this kinase and the consequences of its inhibition on bone metabolism are unknown. To determine if IP6K1 inhibition in obese mice affords protection against obesity-induced metabolic derangements and bone loss, we maintained 2-month-old mice on a normal chow control diet or HFD under thermal neutral conditions for 100 d. Beginning on day 40, HFD-fed mice were divided into two groups and administered daily injections of vehicle or the pan-IP6K inhibitor TNP [N2-(m-Trifluorobenzyl), N6-(p-nitrobenzyl) purine]. HFD-fed mice developed obesity, hyperglycemia, hyperlipidemia, and secondary osteoporosis, while TNP administration protected mice against HFD-induced metabolic and lipid derangements and preserved bone mass, mineral density, and trabecular microarchitecture, which correlated with reduced serum leptin levels, reduced marrow adiposity, and preservation of marrow resident skeletal stem/progenitor cells (SSPCs). TNP also exhibited hypotensive activity, an unrealized benefit of the drug, and its prolonged administration had no adverse impacts on spermatogenesis. Together, these data indicate that the inhibition of IP6K1 using selective inhibitors, such as TNP, may provide an effective strategy to manage obesity and T2DM due to its bone sparing effects.

## 1. Introduction

Obesity and type 2 diabetes mellitus (T2DM) are chronic metabolic diseases with complex pathophysiology. Due to negative impacts of hyperglycemia and excessive body fat on bone metabolism, both conditions are also associated with an increased risk for low-trauma fragility fractures and fracture complications [1,2]. For example, hyperglycemia drives the accumulation of advanced glycation end products that impair bone formation and decrease bone strength by inducing excessive non-enzymatic cross-linking of the collagen matrix [3]. A strong correlation also exists between increased body fat mass and marrow adipose tissue (MAT) volume and bone loss in mouse models of DIO [4,5,6,7] due to the anti-osteogenic activity of secreted fatty acids, adipokines, and RANKL [8,9,10,11]. Excess body fat has also been linked to high serum leptin levels [12,13], and while effects of leptin on bone are complex [14,15], its overproduction is associated with a low bone mass phenotype [16]. Obesity-induced increases in serum leptin levels have also been shown to skew bifurcation of leptin receptor (LEPR) expressing skeletal stem/progenitor cells (SSPCs), which function as precursors of bone and fat in adult bone marrow [17], toward adipogenesis at the expense of osteogenesis [18]. Managing bone loss in T2DM patients is complicated by the fact that many anti-diabetic drugs, including thiazolidinediones, incretin-based therapies, and sodium-glucose co-transporter 2 inhibitors, promote MAT accumulation at the expense of skeletal mass, which limits their overall effectiveness [19,20,21,22,23,24,25,26]. Consequently, there remains a pressing need to identify novel anti-diabetic medications that exhibit beneficial impacts on skeletal health.

Inositol pyrophosphates are a family of signaling molecules that play important roles in phosphate sensing, cellular energy regulation, insulin signaling, and metabolic disease [27,28,29]. These compounds are synthesized by a family of three inositol (Ins) hexakisphosphate kinases (IP6K1-3) that generate InsP7 from InsP6. Epidemiologic data have linked IP6K1, the major IP6K isoform, to obesity in humans [30], and genetics-based studies have identified the kinase as a critical driver of metabolic disease, insulin resistance, and fatty liver in rodent models of DIO. For example, DIO mice harboring a global deletion of *Ip6k1* (*Ip6k1^−/−^*) exhibit enhanced insulin and leptin sensitivity and resistance to HFD-induced weight gain due to increased AKT signaling [31], and the adipocyte-specific deletion of *Ip6k1* yields similar effects by increasing thermogenic energy expenditure and AMPK activity [32]. The pharmacological inhibition of IP6K1 using the pan-IP6K inhibitor TNP also yields potent anti-diabetic effects in obese mice. [33] However, the impacts of *Ip6k1* knockout and TNP administration in mouse models of DIO are diminished but not abolished under thermoneutral conditions, [33,34] which eliminates the impacts of increased energy expenditure in response to mild cold challenge. These results indicate that the protective effects of IP6K1 are only partially dependent on environmental temperature, which itself is an important dependent variable in DIO studies. Despite its potent anti-diabetic effects, no studies have evaluated how IP6K1 inhibition impacts skeletal integrity and marrow adiposity in models of DIO.

In this paper, we show that TNP administration after onset of HFD feeding protects mice from DIO and obesity-induced bone loss under thermo-neutral conditions by preventing HFD-induced increases in body weight, body fat mass, serum leptin levels and MAT volume. TNP administration also prevented HFD induced decreases in the number of SSPCs in bone marrow, and stimulated osteogenesis of human MSCs. Together, these results are consistent with previous reports showing that TNP administration protects mice from DIO and extend these findings by demonstrating it preserves bone mass and microarchitecture and prevents the expansion of marrow adipose tissue (MAT) volume in HFD-fed mice. By doing so, they identify IP6K1 as a potential therapeutic target for glycemic control due to its positive impacts on bone health.

## 2. Materials and Methods

### 2.1. Cell and Cell-Based Assays

Human MSCs were enriched from bone marrow aspirates of the iliac crest as described previously [35]. Donor populations were plated at 25,000 cells per well in 48-well plates in complete culture media (CCM; α-MEM supplemented with 16.5% FBS, 2 mM L-glutamine, 100 units/mL penicillin and 100 μg/mL streptomycin). After 2–3 d, media was replaced with 0.5 mL of osteo-inductive media (low glucose DMEM supplemented with 10% FBS, 100 nM Dexamethasone, 10 mM β-glycerolphosphate, 50 μg/mL L-ascorbic acid 2-phosphate, 100 units/mL penicillin and 100μg/mL streptomycin) or adipo-inductive media (CCM supplemented with 0.5 μM dexamethasone, 0.5 mM isobutylmethylxanthine, and 50 μM indomethacin). Culture media and supplements were purchased from GIBCO and FBS was obtained from Sigma (St. Louis, MO, USA). Cultures were also supplemented with 0.5 uL of TNP (10 mg/L) or DMSO:Tween80:water (1:1:8), which was used as vehicle. Media were replaced every 5–7 d and after a total of 3 weeks the extent of osteogenic differentiation was quantified by fixing cells in neutral buffered formalin (0.25 mL), washing plates 3x in PBS and staining with Alizarin Red S (0.2 mL/well; Sigma) for 30 min at room temperature. After extensive washing, dye was extracted with 10% (*w*/*v*) cetylpyridinium chloride (Sigma) in 10 mM sodium phosphate, pH 7.0 for 15 min at room temperature and quantified spectroscopically at 562 nm. Adipogenic differentiation was quantified by staining monolayers with AdipoRed (0.2 mL; Sigma) for 10 min at room temperature, washing plates with PBS, and directly quantifying the dye spectroscopically at 572 nm. Absorbance measurements were made using a Synergy^TM^ HT Multi-Mode Reader. 

### 2.2. Mice and Experimental Design

All animal studies were approved by the Institutional Animal Care and Use Committee of UF Scripps Biomedical Research. Two-month-old C57BL/6 mice (The Jackson Laboratory, Bar Harbor, ME, USA) housed in controlled light and temperature conditions were divided into two groups based on body weight (NC = 26.9 ± 1.9; HFD = 26.7 ± 1.6; *p* = 0.72) to eliminate bias in bone mass measurements due to body weight differences, and fed normal chow (NC; 16%, 60% and 24% calories from fat, carbohydrate and proteins, respectively) or a high-fat diet (#D12451, Research Diets, New Brunswick, NJ, USA) that consisted of 45% calories from fat (HFD; 45%, 35%, and 20% calories from fat, carbohydrate, and protein, respectively) for 2 wk at 23 °C. Mice were then acclimatized to housing at thermo-neutral temperature (32 °C) and maintained on NC (n = 8–10 mice/group) or HFD (n = 16–20 mice/group) for a total of 100 days. After 40 d, HFD-fed mice were randomly divided into two groups (n = 8–10 mice/group) and administered TNP (10 mg/kg, daily, IP) or vehicle (DMSO:Tween80:water, 1:1:8). Body weight was measured weekly, and body composition was quantified by QNMR at the onset of HFD feeding and at the study endpoint. Blood draws were performed at the study endpoint prior to euthanasia. Metabolic profiling was conducted in the Metabolic Core at UF Scripps Biomedical Research. 

### 2.3. Chemicals

N2-(m-Trifluorobenzyl), N6-(p-nitrobenzyl)purine (TNP) was synthesized as described previously and the structure confirmed by NMR [33]. 

### 2.4. Micro-Computed Tomography

Femurs and tibiae were collected at the study endpoint and analyzed by micro-CT using the µCT35 system (SCANCO Medical AG) with an X-ray source operating at a beam energy of 70 kVp and 113 μA. Briefly, scans (7μ resolution, 1000 projections/180°) of trabecular bone in the proximal tibia consisted of 300 slices covering 2.1 mm from the growth plate, and scans of cortical bone consisted of 57 slices covering 0.4 mm of the tibia mid-shaft. Segmentation of trabecular bone images was conducted on 200 total slices beginning 10 slices from the growth plate following manual contouring (gray-scale threshold of 240–1000 using a permille scale equivalent to 3313 Hounsfield units). Segmentation of cortical bone images was conducted on the entire image stack of 57 slices following semi-automatic contouring (3673 Hounsfield Units). In both cases, the Gauss (3D) noise filter was set to sigma 1.2 and support 1.0. Trabecular and cortical bone morphometric parameters were calculated directly from voxel values and included TV, BV, Tb.N, Tb.Th, Tb.Sp, Conn.D, SMI, Ct.Th, M.Ar, and BMD. Analysis of lipid distribution and volume was performed on decalcified bone specimens stained for 2 h in a solution containing 2% osmium tetroxide prepared in 0.1 M sodium cacodylate buffer (pH 7.4) and images of lipid deposits acquired at 12 µ resolution with 500 projections/180°, a gray-scale threshold of 480–1000, and with Gauss noise filter at sigma 1.2 and support 2.0. Lipid volumes were calculated directly from voxel volumes for the tibia and femur. 

### 2.5. Fluorescence-Activated Cell Sorting

Bone marrow plugs recovered from tibiae and femurs of mice (n = 6–9) from each experimental group were incubated in digestion buffer containing 500 µg/mL LiberaseDL^®^ at 37 °C in three intervals of 15 min to release SSPCs. Marrow cells were filtered using a cell strainer (40 µM) to remove debris and then stained with propidium iodide to assess viability. Cells were also stained with antibodies against the lineage markers TER-119 (Tonbo Biosciences, Cat #: 35–5921, clone #: TER-119), CD31 (BD Pharmingen, Cat #: 553372, clone #: MEC13.3) and CD45 (Tonbo Biosciences, Cat #: 35–0451, clone #: 30-F11), biotinylated anti-LEPR antibody (R&D Systems, Cat # BAF497) and BV421 Streptavidin (BD Biosciences, Cat #: 563259). Thereafter, the Lin^−^/LEPR^+^ fraction was sorted using a BD FACSAria3 flow cytometer in the UF Scripps Biomedical Research Flow Cytometry Core using a gating strategy that excludes doublets and non-viable cells.

### 2.6. Spermatogenesis

Reproductive organ dissection, sperm collection, and analysis of sperm morphology were performed as previously described [36]. Briefly, at the study endpoint, testes and epididymis were dissected from euthanized HFD mice treated with vehicle or TNP and weighed (n = 6–7 mice/group). Sperm were expressed from the cut ends of cauda epididymis (n = 6–7 mice/group) and viability and morphology were analyzed. Daily sperm production (DSP) was also quantified.

### 2.7. Statistical Analysis

All statistical analysis were performed using GraphPad Prism 9. All data are reported as mean ± standard deviation. The statistical significance between two independent experimental groups was assessed using a two-tailed, unpaired Student’s *t*-test and that for more than two groups was assessed using a one-way ANOVA with multiple comparisons assessed using the Tukey’s test for groups with equal sample sizes, and the Tukey–Kramer test for groups with unequal sample sizes. Significance levels were set at *p* ≤ 0.05. 

## 3. Results

### 3.1. TNP Administration Reverses HFD-Induced Weight Gain and Hyperglycemia

**Figure 1:**
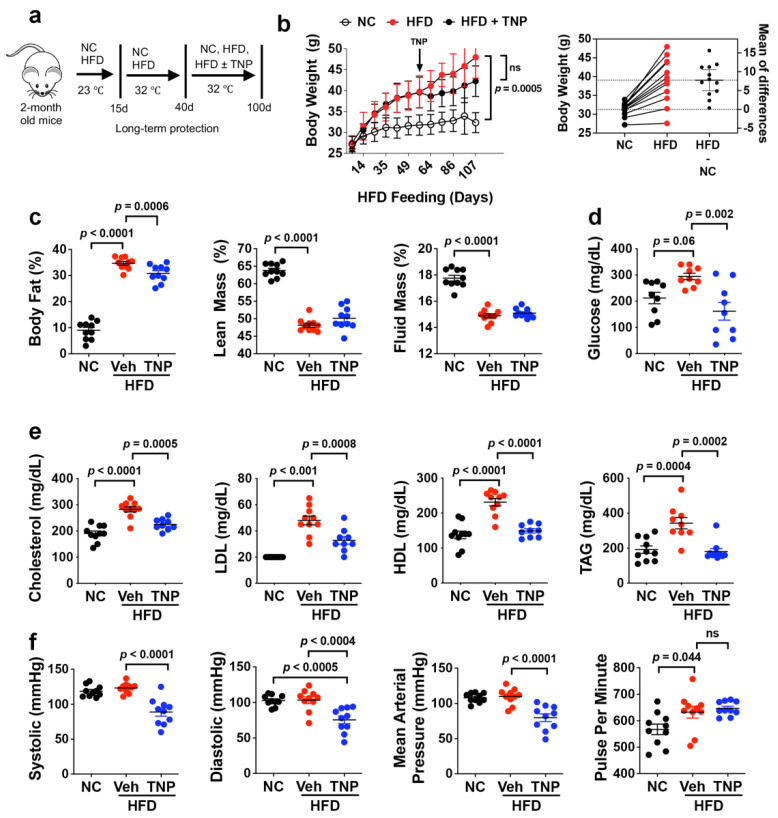
TNP protects mice from HFD-induced weight gain, hyperglycemia, and hyperlipidemia. (**a**) Schematic of the feeding regimen and timing of daily TNP administration (10 mg/kg BW, IP). NC, normal chow; HFD, high-fat diet. (**b**) Mouse body weights measured at weekly intervals over the feeding time course and estimation plot of vehicle and TNP-treated HFD mice showing raw data (**left**) and the group means difference with 95% confidence interval (**right**). (**c**) Baseline and endpoint measures of body fat, lean mass, and fluid mass in mice from (**a**). (**d**,**e**) Endpoint measure of blood glucose levels (**d**) and serum cholesterol, LDL, HDL, and TAG levels (**e**) in mice from (**a**). (**f**) Endpoint measures of systolic, diastolic, and mean arterial pressure and pulse rate of mice in (**a**). Data are mean ± SD (n = 8–14 mice/group). In Prism 9, *p*-values are by one-way ANOVA and Tukey’s post hoc test for (**b**–**f**).

To assess impacts of IP6K1 inhibition on obesity-induced bone loss, we subjected 2-month-old mice to HFD feeding for 100 d under thermo-neutral conditions to prevent adaptive thermogenesis and after 40 d administered daily injections of vehicle or TNP (10 mg/kg BW) [37] (Figure 1a). As anticipated, vehicle-treated HFD-fed mice gained significantly more weight over the feeding time course as compared to the age- and sex-matched NC-fed controls. Moreover, while the body weights of HFD-fed mice given vehicle vs. TNP were not significantly different over the study time course, weight gain was blunted in HFD-fed mice after the onset of TNP administration resulting in ~6 g decrease in average body weight by the study endpoint (Figure 1b). HFD-fed mice also exhibited significant increases in endpoint measures of body fat mass and significant decreases in lean body and fluid mass (Figure 1c) as compared to NC-fed mice, while TNP administration significantly reduced body fat mass in HFD-fed mice but had no effect on lean body and fluid mass (Figure 1c). Blood profiling further showed that HFD feeding resulted in significant increases in blood glucose (Figure 1d) and various lipid markers, including cholesterol, LDL, HDL, and triacylglycerol (TAG) (Figure 1e), as compared to the NC-fed controls, and that TNP administration significantly reversed HFD-induced hyperglycemia and hyperlipidemia (Figure 1d, e). These results are consistent with a previous studying showing that TNP prevents weight gain and restores metabolic homeostasis in mice when administered after the onset of HFD feeding [33]. Unrelated to its effects on metabolism, we also observed that TNP administration caused a significant reduction in systolic, diastolic, and mean arterial blood pressure in HFD-fed mice without significantly affecting pulse rate (Figure 1f). The hypotensive activity of TNP in HFD mice represents a previously unrealized benefit of this drug.

### 3.2. TNP Administration Prevents HFD-Induced Bone Loss and MAT Accumulation

**Figure 2:**
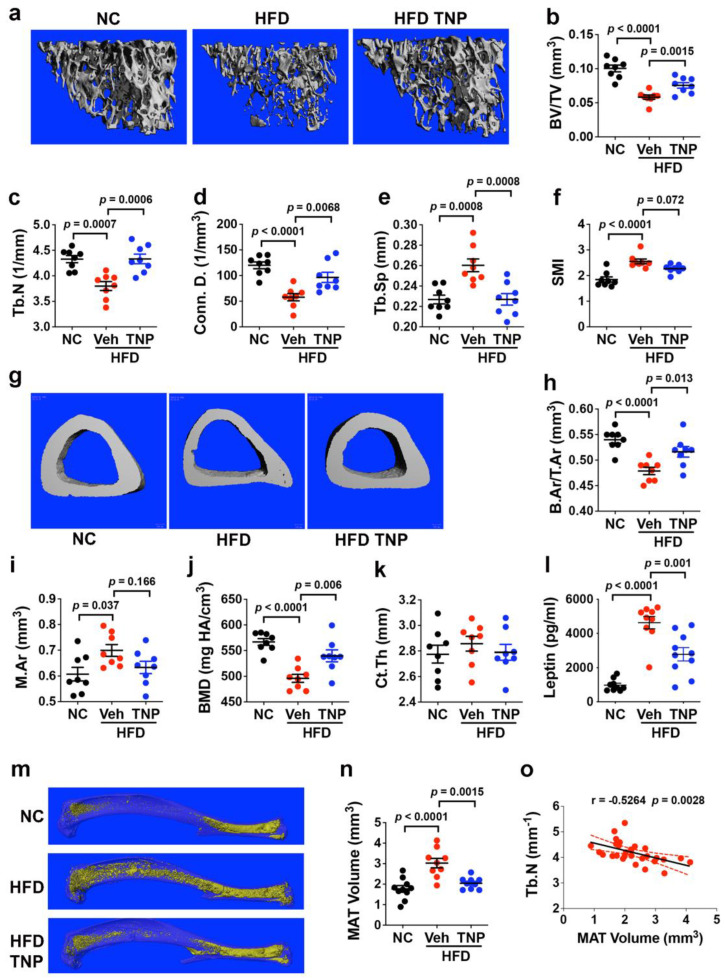
TNP prevents obesity-induced bone loss. (**a**,**g**) Representative micro-CT images of the proximal (**a**) and midshaft (**g**) tibiae of mice maintained on NC or HFD with or without TNP administration. (**b**–**f**) Quantification of BV/TV (**b**), Tb.N (**c**), Conn. D (**d**) and Tb.Sp (**e**), and SMI (**f**) by micro-CT. (**h**–**k**) Quantification of B.Ar/T.Ar (**h**), M.Ar (**i**), BMD (**j**), and Ct. Th (**k**) by micro-CT. (**l**) Quantification of plasma leptin levels by ELISA. (m) Representative micro-CT images of marrow adiposity in tibiae stained with osmium tetroxide. (n) Quantitation of MAT volume by micro-CT in bones from (**m**). (**o**) Scatter plot of Tb. N vs. MAT volume for all mice. Data are mean ± SD (n = 8–10 mice/group). In Prism 9, *p*-values are one-way ANOVA and Tukey’s post hoc test for (**b**–**f**,**h**–**l**,**n**,) and Pearson’s correlation coefficient and corresponding *p*-value for (**o**).

To quantify impacts of HFD feeding and IP6K1 inhibition on skeletal pathology, we analyzed tibiae recovered from mice at the study endpoint by micro-CT. The analysis of the proximal tibiae (Figure 2a) revealed that HFD vs. NC mice exhibited significant decreases in trabecular bone volume as a percentage of total volume (BV/TV) (Figure 2b). With respect to trabecular microarchitecture, trabecular number (Tb.N) (Figure 2c) and connectivity density (Conn.D) (Figure 2d) were significantly decreased, while trabecular spacing (Tb.Sp) (Figure 2e) was significantly increased in HFD- vs. NC-fed mice. Structure model index (SMI) was also significantly increased (Figure 2f), indicating that trabecular bone loss in HFD mice resulted from generalized thinning of trabeculae plate-like to rod-like structures [38]. These negative impacts of HFD feeding on trabecular bone volume and microarchitecture were reversed to a significant extent by TNP administration (Figure 2b–d), which also resulted in a measurable but non-significant (*p* = 0.072) decrease in SMI (Figure 2f). Analysis of the tibiae midshaft (Figure 2g) further revealed that HFD vs. NC mice exhibited decreased cortical bone area as a fraction of total area (B.Ar/T.Ar) (Figure 2h), increased total marrow area (M.Ar) (Figure 2i), and decreased cortical bone mineral density (BMD) (Figure 2j), while no significant change in cortical thickness (Ct. Th) was observed (Figure 2k). These impacts of HFD feeding are consistent with studies showing that obesity/T2DM alters cortical BMD by increasing porosity without altering thickness, thereby negatively impacting overall structural integrity [39,40]. HFD-induced alterations in the cortical bone area and density were also largely reversed in response to TNP administration (Figure 2h–k). Although TNP-mediated weight loss was shown to be blunted at thermoneutral conditions [33], our studies demonstrate that the drug afforded significant protection against obesity-induced bone loss in mice in the absence of adaptive thermogenesis.

HFD feeding also induced significant increases in serum leptin levels (Figure 2l), and the micro-CT analysis of osmium tetroxide-stained tibiae (Figure 2m) showed it also promoted the significant expansion of MAT volume (Figure 2n). In contrast, HFD-fed mice administered TNP exhibited significantly lower serum leptin levels and MAT volume as compared to their vehicle-treated counterparts. Consistent with the known negative impacts of MAT on bone health, we also observed a highly significant negative correlation between MAT volume and Tb. N. among all mouse cohorts (Figure 2o). These data indicate that the bone sparing activity of TNP results, in part, by its ability to prevent the expansion of visceral and bone marrow fat depots, which contribute to obesity-induced bone loss via systemic and localized impacts on bone metabolism.

### 3.3. TNP Administration Preserves the SSPC Pool in the Bone Marrow of HFD-Fed Mice

**Figure 3:**
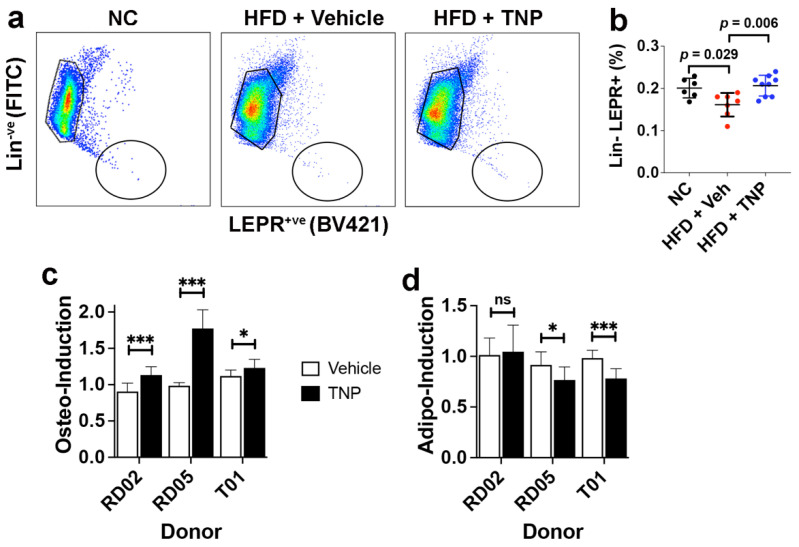
TNP administration prevents the HFD-induced contraction of the SSPC pool in the bone marrow. (**a**) Representative dot plots from flow cytometric analysis of whole bone marrow cells stained with antibodies against lineage-specific markers (CD31, CD45, and Ter119) and LEPR. (**b**) Percentage of Lin^−^LEPR^+^ SSPCs recovered from the bone marrow of mice fed NC or HFD and treated with vehicle or TNP. Data are mean ± SD (n = 6–9 mice/group). (**c**,**d**) Extent of osteo-induction (**c**) and adipo-induction (**d**) of human MSCs from the indicated donors when cultured in vehicle vs. TNP (10 mg/L) over a 3-week time course of cellular differentiation. Data are mean ± SD from biological replicates performed in quadruplicate. In Prism 9, *p*-values are by one-way ANOVA and Tukey’s post hoc test for (**b**) and Student’s *t*-test (vehicle vs. TNP) for c and d with * *p* < 0.05, *** *p* < 0.005, ns = not significant.

Based on a recent study reporting a significant reduction in the percentage of bone marrow cells with colony-forming unit-fibroblast activity in HFD mice [18], the majority of which derive from the Lin^−^LEPR^+^ fraction of SSPCs [17], we questioned if TNP administration impacted the size of this SSPC pool in bone marrow. Therefore, we enriched the Lin^−^LEPR^+^ cell fraction from the bone marrow of NC and HFD mice administered vehicle or TNP via FACS (Figure 3a). This analysis revealed that Lin^−^LEPR^+^ SSPC frequency was significantly lower in HFD- vs. NC-fed mice, while SSPC frequency was not significantly different in HFD-fed mice administered TNP as compared to NC-fed controls (Figure 3b). Additionally, we stimulated bone-marrow-derived MSCs obtained from three separate human donors to undergo osteogenic and adipogenic differentiation in the absence or presence of TNP (10 mg/L). While we observed inter-donor differences in drug sensitivity, TNP significantly augmented the extent of osteogenic differentiation of all three donors (Figure 3c) and significantly inhibited adipogenic differentiation in two of three donors (Figure 3d). These findings are consistent with previous data showing that primary MSCs from the bone marrow of *Ip6k1*^−/−^ vs. wild-type mice exhibit improved fitness and increased osteogenic potential at the expense of adipogenesis [41]. Together, these results demonstrate that, in addition to protecting mice against obesity-induced bone loss, TNP administration also preserves the pool of SSPCs in the bone marrow and skews bifurcation in favor of osteogenesis at the expense of adipogenesis.

### 3.4. TNP Has No Long-Term Negative Impacts on Spermatogenesis in Adult Male Mice

**Figure 4:**
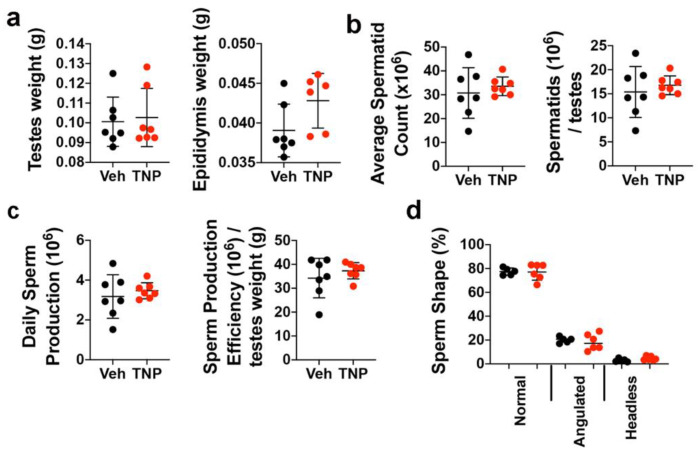
Prolonged TNP administration does not impair spermatogenesis in male mice. (**a**) Weight of testes and epididymis from vehicle- or TNP-treated HFD mice. (**b**) Average spermatid counts and spermatid concentrations per testes. (**c**) Daily sperm production and sperm production efficiency. (**d**) Percentages of sperm with normal morphology or abnormalities, including angulated midpieces and headless tails. Data are mean ± SD (n = 6–7 mice/group). In Prism 9, *p*-values are by Student’s *t*-test.

Bhandari et al. [42] previously showed that male *Ip6k1^−/−^* mice are infertile due to a significant reduction in the number of spermatids in the seminiferous tubules and absence of sperm in the epididymis. Therefore, to determine whether, if prolonged, daily administration of TNP yielded any long-term impacts on fertility in adults, we compared spermatogenesis in HFD male mice after 60 d of TNP or vehicle administration. We observed no significant difference in testes and epididymis weights (Figure 4a) and in average total spermatid counts or the total number of spermatids per testis (Figure 4b) between treatment groups. Functional tests also failed to reveal any significant differences in daily sperm production or the efficiency of sperm production as a function of testes weight (Figure 4c) between treatment groups, and TNP administration also had no adverse effects on sperm morphology (Figure 4d). These findings are consistent with a previous study showing that NC-fed male mice administered TNP for 15 weeks were capable of impregnating female mice [33]. Since DIO decreases fertility in male mice due to alterations in testicular morphology, decreased sperm motility, and alterations in sex hormone levels [43,44], these results provide important evidence that TNP has no negative impacts on reproductive parameters when administered long-term to male DIO mice. Evaluating fertility vs. toxicity is important since obese young male adults may benefit from IP6K1 inhibition.

## 4. Discussion

HFD feeding is an accepted model of obesity in mice, but the genetic background, age, sex of animals, duration of feeding, and thermoregulatory characteristics influences the extent of bone loss [45,46]. In this paper, we demonstrated that male mice housed under thermoneutral conditions exhibit significant weight gain, hyperglycemia, hyperlipidemia, increased marrow adiposity, and secondary osteoporosis in response to HFD feeding, and that the daily administration of the pan-IP6K inhibitor TNP affords protection against HFD-induced metabolic derangements, resulting in a significant preservation of bone volume, density, and microarchitecture. Although TNP’s weight loss effects are dependent on environmental temperature and are less potent at thermoneutrality, we found that the drug still afforded a significant protection against obesity-induced bone loss in DIO mice maintained under thermoneutral conditions. While our data do not distinguish whether TNP preserves bone health due to direct effects on bone formation and/or indirect effects on weight gain and metabolism, we previously showed that the yields of MSCs were significantly higher from the bone marrow of *Ip6k1^−/−^* vs. wild-type mice, and that *Ip6k1^−/−^* MSCs exhibited superior hematopoiesis-supporting activity and bone forming activity and reduced adipogenic differentiation as compared to *Ip6k1^+/+^* MSCs, which was linked mechanistically to decreased p53 protein expression and lower mitochondrial ROS production^41^. Studies demonstrating that TNP also skews bifurcation of human MSCs in favor of osteogenesis at the expense of adipogenesis are consistent with our findings in mice and implicate IP6K1 in directly influencing MSC fitness and fate determination. Studies showing that TNP also prevents DIO-induced decreases in the frequency of Lin^−^LEPR^+^ SSPCs in bone marrow are consistent with these data. However, since this effect may also be direct and related to the preservation of trabecular microarchitecture, additional studies are needed to verify if IP6K1 has cell-autonomous dependent effects in SSPCs. Notably, TNP was initially identified in a screen for purine-based inhibitors of IP3-3K [47] and thereafter was shown to have ~70-fold greater potency for IP6K1 and dose-dependently reduce InsP7 levels by >90% in cells without affecting levels of other inositol phosphates [37]. Therefore, TNP functions as a relatively selective and reversible inhibitor of IP6Ks. Although TNP also inhibits IP6K2 and IP6K3, data showing that its anti-diabetic effects were abolished in *Ip6k1^−/−^* mice confirm the drugs selectivity in vivo [33]. Therefore, based on activities reported for TNP by us and others, the development of more highly selective and potent IP6K1 inhibitors is anticipated to yield novel with more potent anti-diabetic and bone-sparing activities.

## 5. Conclusions

Studies showing that TNP protects mice against DIO are consistent with the drugs known ability to enhance thermogenic energy expenditure. Additionally, they further demonstrated that TNP has potent bone-sparing effects in DIO mice even under thermoneutral conditions and expand its range of functions to include hypotensive activity in HFD-fed mice and pro-osteogenic activity in cell-based assays. They also confirm that the long-term administration of TNP had no effect on spermatogenesis. Therefore, unlike therapeutic interventions that target PPARG and LEPR activity, which yield detrimental side effects including increased fracture risk and altered feeding behavior, respectively, the inhibition of IP6K1 maintains insulin sensitivity and prevents obesity while preserving bone integrity. Together, these properties validate IP6K1 as a preferred drug target for the treatment of diabetic bone and, as such, warrant the identification of more potent and selective IP6K1 small-molecule inhibitors. 

## Data Availability

The data presented in this study are available on request from the corresponding author.
